# Macrophage cytotherapy on liver cirrhosis

**DOI:** 10.3389/fphar.2023.1265935

**Published:** 2023-12-15

**Authors:** Dabing Ping, Yuan Peng, Xudong Hu, Chenghai Liu

**Affiliations:** ^1^ Institute of Liver Diseases, Shuguang Hospital Affiliated with Shanghai University of Traditional Chinese Medicine, Shanghai, China; ^2^ Department of Biology, School of Integrative Medicine, Shanghai University of Traditional Chinese Medicine, Shanghai, China; ^3^ Shanghai Key Laboratory of Traditional Chinese Clinical Medicine, Shanghai, China; ^4^ Key Laboratory of Liver and Kidney Diseases, Ministry of Education, Shanghai, China

**Keywords:** cell therapy, liver cirrhosis, macrophage cytotherapy, mechanism, transfusion

## Abstract

Macrophages, an essential cell population involved in mediating innate immunity in the host, play a crucial role on the development of hepatic cirrhosis. Extensive studies have highlighted the potential therapeutic benefits of macrophage therapy in treating hepatic cirrhosis. This review aims to provide a comprehensive overview of the various effects and underlying mechanisms associated with macrophage therapy in the context of hepatic cirrhosis.

## 1 Introduction

Liver cirrhosis is a serious health concern that affects people worldwide, with over one million deaths occurring annually due to end-stage liver disease. China alone accounts for 50% of the cases (Asrani et al., 2019). Given the high mortality rate associated with this disease, researchers have been focusing on inhibiting or reversing cirrhosis. In recent years, macrophage infusion therapy has emerged as a promising treatment modality for liver disease. Macrophages are immune cells that are highly abundant in the liver, and there is growing evidence to suggest that macrophage infusion therapy can help reverse cirrhosis. This review provides an overview of the latest research on macrophage infusion therapy, which has shown great potential for treating liver fibrosis and improving development prospects.

## 2 Diversity and plasticity of hepatic macrophages

Hepatic macrophages make up a significant portion of all macrophages in the body and are composed of both liver-resident and various infiltrated macrophages. Normally, liver-resident Kupffer cells (KCs), which originate from the yolk sac, comprise the primary population of hepatic macrophages. When the liver is injured, bone marrow monocytes are recruited to the liver and acquire the function of KCs (Borst et al., 2018; Peiseler et al., 2023).

Macrophages are a highly versatile and diverse group of immune cells that are widely distributed throughout the body. Traditionally, macrophages can be classified into two primary types based on their activation pathways, biomarkers, and cytokine release: classically activated type 1 macrophages (M1) and alternatively activated type 2 macrophages (M2). M1 macrophages are activated through lipopolysaccharides and IFN-gamma, producing an abundance of proinflammatory cytokines such as IL-1β, IL-6, and TNF-α to mediate inflammation. In contrast, M2 macrophage formation is stimulated by cytokines such as IL-4, IL-10, and IL-13, which promote tissue remodeling while suppressing inflammation (Mosser and Edwards, 2008; Murray and Wynn, 2011). Given the enormous heterogeneity of macrophages, a new naming approach is proposed that describes the stimulus scenario and adopts nomenclature related to activation standards, which can to some extent alleviate the complexity of macrophages, i.e., M (IL-4), M (Ig), M (IL-10), M (GC), M (IFN-γ), M (LPS) and so forth (Murray et al., 2014).

In mouse experiments, bone marrow-derived macrophages express high levels of lymphocyte antigen 6 complex locus C (Ly-6C) (Mossanen et al., 2016) These macrophages also have the potential to differentiate into Ly-6C low expression cells, which demonstrate high levels of matrix metalloproteinases (MMPs) capable of degrading collagen (Lu et al., 2020). However, Ly-6C^high^ and Ly-6C^low^ cells do not correspond one-to-one with M1 and M2 macrophages, they may represent a phenotype that is intermediate between the two (Ramachandran et al., 2012). There are no antigens in the human body that correspond to Ly-6C, but it is often suggested that Ly-6C^high^ and Ly-6C^low^ monocytes are functionally analogous to human CD14^+^CD16^−^ and CD14^+^CD16^+^ monocytes, respectively (Liaskou et al., 2013). However, CD14^+^CD16^+^ monocytes exhibit both pro-inflammatory and phagocytic activities. The former has opposing effects compared to Ly-6C^low^ cells, while the latter exhibits similar characteristics (Ramachandran et al., 2012; Liaskou et al., 2013).

## 3 Macrophage therapy on liver cirrhosis

The wide-ranging adaptability of macrophages underscores their significant contribution to hepatic fibrosis. In mice, Kupffer cell levels experienced rapid depletion during early-stage partial hepatectomy but recovered gradually thanks to local proliferation and infiltrating monocyte-derived macrophage replenishment (Ma et al., 2022). The restoration of macrophage function varied significantly at different stages of liver regeneration (Elchaninov et al., 2021). Furthermore, the absence of hepatic macrophages significantly hindered liver recovery from acetaminophen-induced liver injury (You et al., 2013). Studies with CD11b-DTR transgenic mice showed that deleting infiltrating macrophages during fibrosis regression impeded extracellular matrix (ECM) degradation, which worsened fibrosis (Duffield et al., 2005). Past research with Ccr2^−/−^ transgenic mice affirmed the findings and strongly suggested that infiltrating macrophages play a pivotal role in resolving hepatic damage (Hogaboam et al., 2000; Dambach et al., 2002).

Liver cirrhosis was previously considered a troublesome disease, but recent studies have suggested that mesenchymal stem cell therapy may be able to induce cirrhosis reverse (Shi et al., 2021). Additionally, exogenous macrophage transplantation therapy, which can be autologous, allogeneic, or syngeneic, represents an alternative technique for regulating the hepatic microenvironment and shows promise in the treatment of liver fibrosis. Table 1 provides an overview of some of the promising studies that have been conducted on macrophage infusion therapy.

**TABLE 1:** Macrophage transplantation therapy for liver disease.

Study	Liver diseases models	Macrophage modulation	Injection times	Treatment times	Dose	Route	Results	Intercellular crosstalk	Mechanisms
Ke et al. (2010)	Hepatic ischemia reperfusion	modified HO-1 expressing BMDMs	24 h prior to the ischemia	-	5 × 10^6 cells	infusion via tail vein	ameliorate IR-mediated local organ damage	hepatocyte, neutrophil	-
Thomas et al. (2011)	CCl_4_ induced liver fibrosis	BMDMs	24 h after the 12th injection (6 weeks)	4 weeks	1 × 10^6 cells	infusion via portal vein	improved liver fibrosis	macrophages, neutrophils, HSCs	Decreasing HSCs amount; Promote ECM degradation; Recruitment of endogenous macrophages; stimulate regenerative response
Bird et al. (2013)	Normal mice	BMDMs	-	21 days	1 × 10^7 cells	infusion via tail vein	activate ductular reaction	hepatic ductular cells	dependent on TWEAK signaling
Huang et al. (2014)	Hepatic ischemia reperfusion	modified HO-1 expressing BMDMs	24 h prior to the ischemia	-	5 × 10^6 cells	infusion via portal vein	ameliorate IR-mediated injury	hepatocyte, neutrophil	-
Moore et al. (2015)	CCl_4_ induced liver fibrosis	HMDMs	24 h after the 12th injection (6 weeks)	4 weeks	5 × 10^6 cells	infusion via the spleen	improved liver fibrosis	HSCs	suppress HSCs activation
Ma et al. (2017)	CCl_4_/Bile duct ligation operation induced liver fibrosis	BMDMs, BMDMs (LPS/INF-γ), BMDMs (IL-4) macrophages	24 h after the 8th injection (4 weeks)/after 10 days BDL operation	4 weeks/11 days	1 × 10^6 cells	infusion via the tail vein	Both BMDMs, BMDMs (LPS/INF-γ) improved liver fibrosis, the latter performs strongly	HSCs, macrophages, hepatocytes; NK cells	Promoted HSCs apoptosis by amplifying NK cell activation; Promote ECM degradation; Recruitment of endogenous macrophages; improved hepatocyte proliferation

Study	Liver diseases models	Macrophage modulation	Injection times	Treatment times	Dose	Route	Results	Intercellular crosstalk	Mechanisms
Haideri et al. (2017)	CCl_4_ induced liver fibrosis	ESDMs	start of the second week	3 weeks	10 or 20 10^6 cells	infusion via portal vein	low number was no significant, whereas twice significant improved liver fibrosis	hepatic ductular cells, HSCs	Decreasing HSCs amount; Increasing the number of hepatic ductular cells
Yang et al. (2020)	Orthotopic liver transplantation	BMDMS (dexamethasone)	follow OLT	7 days	3 × 10^6 cells	infusion via portal vein	alleviated liver acute rejection	CD8^+^ T cells	Reduced the CD8^+^ T cells
Li and He (2021)	CCl_4_ induced liver fibrosis	Kuffer cells	24 h after the 8th injection (4 weeks)	4 weeks	1 × 10^6 cells	infusion via tail vein	improved liver fibrosis	HSCs, macrophages	Reduced the activations HSCs, Promote ECM degradation
Zhao et al. (2021)	Portal vein ligation	CD86^+^ macrophages	after PVL	1/3/6/7 days	unknown	infusion via portal vein	accelerate liver regeneration	-	-

Abbreviations: IR, ischemia reperfusion; BMDMs, Bone marrow derived macrophages; HSCs, hepatic stellate cells; ECM, extracellular matrix; HMDMs, human monocyte derived macrophages; ESDMs, embryonic stem cell derived macrophages; OLT, orthotopic liver transplantation; PVL, portal vein ligation; LPS, lipopolysaccharides; IFN, interferon; IL, interleukin.

### 3.1 Bone marrow-derived macrophages

Due to the ease of obtaining a large quantity of marrow stem cells, bone marrow-derived macrophages (BMDMs) are a promising candidate. Myelomonocytic cells can differentiate into non-activated macrophages, which can be activated under certain conditions. BMDMs exhibit characteristic macrophage cell surface markers F4/80 and CD11b, and express various mediators including anti-inflammatory, antifibrotic, and chemotactic. In a study using a fibrosis-induced model with carbon tetrachloride (CCl_4_), each mouse was administered 1 × 10^6^ nonpolarized BMDMs through the portal vein. Following cell infusion, a 30% reduction in fibrotic areas was observed after 4 weeks (Thomas et al., 2011). BMDMs activated with lipopolysaccharides and interferon-γ (LPS/IFN-γ) demonstrate increased expression levels of isoform nitric oxide synthase (iNOS), interleukin (IL)-6, IL-12, tumor necrosis factor-alpha (TNF-α), matrix metalloproteinases (MMPs), and macrophage chemokine ligands CCL-2 and CXCL9. In contrast, BMDMs (IL-4) exhibit higher expression levels of arginase-1 and mannose receptor 1 (MRC1), as well as cytokines and chemokines including IL-10, CCL1, and CCL17 (Ma et al., 2017). Macrophages were injected through the tail vein into two different mouse models of chronic liver fibrosis - one induced by toxicity from CCl_4_ and the other from bile duct ligation (BDL) injury. Both BMDMs and BMDMs (LPS/IFN-γ) showed significant improvement in liver fibrosis, and BMDMs (LPS/IFN-γ) exhibited a better therapeutic effect (Ma et al., 2017). Interestingly, BMDMs (IL-4) were found to be ineffective in treating liver fibrosis in this study. However, the adoptive transfer of macrophages stimulated with dexamethasome reduced hepatic markers such as ALT and AST, ameliorating acute rejection in liver transplantation (Yang et al., 2020). This suggests that a specific subpopulation of M2-like macrophages has therapeutic potential for hepatic fibrosis. Additionally, a recent study identified key bioactive lipids that mediate the cytotherapeutic potential of polarized macrophages in post-hepatectomy liver dysfunction. It was found that infusion of BMDMs (IL-4) may have therapeutic potential for post-hepatectomy liver dysfunction (Sun et al., 2021). Overall, BMDMs represent a promising candidate for treating liver fibrosis due to their ability to differentiate into different phenotypes and express various mediators that can regulate the hepatic microenvironment.

### 3.2 Kupffer cells

Kupffer cells (KCs), which are liver-resident macrophages, play a crucial role in regulating liver homeostasis under normal and pathological conditions. In order to investigate the impact of KCs infusion on liver fibrosis, researchers isolated cells directly from the liver. These KCs were found to express CD11b, F4/80, and CD11c, which are markers for monocytes/macrophages (Merlin et al., 2016). It was discovered that the purity of cultured KCs could be improved by using macrophage colony stimulating factor (M-CSF), which also significantly promoted KCs proliferation *in vitro* (Li and He, 2021). Even when expanded *in vitro*, KCs retained their potential polarization and phagocytosis. After 4 weeks of treatment, infusion of KCs through the tail vein was shown to ameliorate liver fibrosis induced by CCl_4_ in mice, indicating that KCs may be another source for macrophage therapy (Li and He, 2021). These findings suggest that KCs may represent a promising target for macrophage therapy in liver fibrosis. By isolating and expanding these cells *in vitro*, it may be possible to generate a large number of high-purity KCs for use in therapeutic applications.

### 3.3 Other specialized macrophages

Large-scale production of therapeutic macrophages is a formidable challenge, but research suggests that induced pluripotent stem cells (iPSCs) could provide a solution (Zhang and Reilly, 2017). Macrophages derived from iPSCs closely resemble monocyte-derived cells in function. Embryonic stem cell-derived macrophages (ESDMs) and bone marrow-derived macrophages (BMDMs) have comparable morphology, with ESDMs appearing slightly larger in size but having lower phagocytic activity than BMDMs (Haideri et al., 2017). Furthermore, ESDMs exhibit lower expression of M1-related genes than BMDMs under conventional polarization regimes, while the expression of M2-related genes is increased. MMP-12 and MMP-13 are also lower in ESDMs compared to BMDMs, while MMP-9 is expressed at a higher level (Haideri et al., 2017). In a study assessing the antifibrotic capacity of ESDMs, cells were infused via intravenous injection 1 week after CCl_4_ injections, and analysis was conducted 21 days later. 50% reduction of fibrosis level was achieved when injecting 20 × 10^6^ ESDMs, but there was no significant effect on fibrosis levels when using half the number of ESDMs. Notably, ESDMs were found to be more effective than BMDMs in repopulating Kupffer cell-depleted livers due to their phenotype being more similar to tissue resident macrophages (Haideri et al., 2017). These results highlight the importance of carefully considering the dosage and timing of ESDM delivery in order to maximize its potential therapeutic benefits. Despite requiring a larger number of ESDMs to achieve efficacy, they were still able to replicate the regression of fibrosis induced by primary macrophage infusion.

Primary human monocyte-derived macrophages (HMDMs) obtained from healthy donors exhibit a notable increase in the phagocytosis and repair markers CD163, CD169, and CD206, while showing a decrease in the inflammatory cytokine receptor CCR2. The transplantation of HMDMs via the spleen into an immunocompromised mouse model of liver fibrosis resulted in a regression of collagen and a reduction in markers of liver injury. These findings provide good manufacturing practice (GMP) -compatible protocols for large-scale manufacturing to produce HMDMs of clinical grade, which may be a promising source for macrophage therapy in liver fibrosis (Moore et al., 2015).

## 4 The mechanism underlying macrophage therapy on mouse liver fibrosis

**FIGURE 1:**
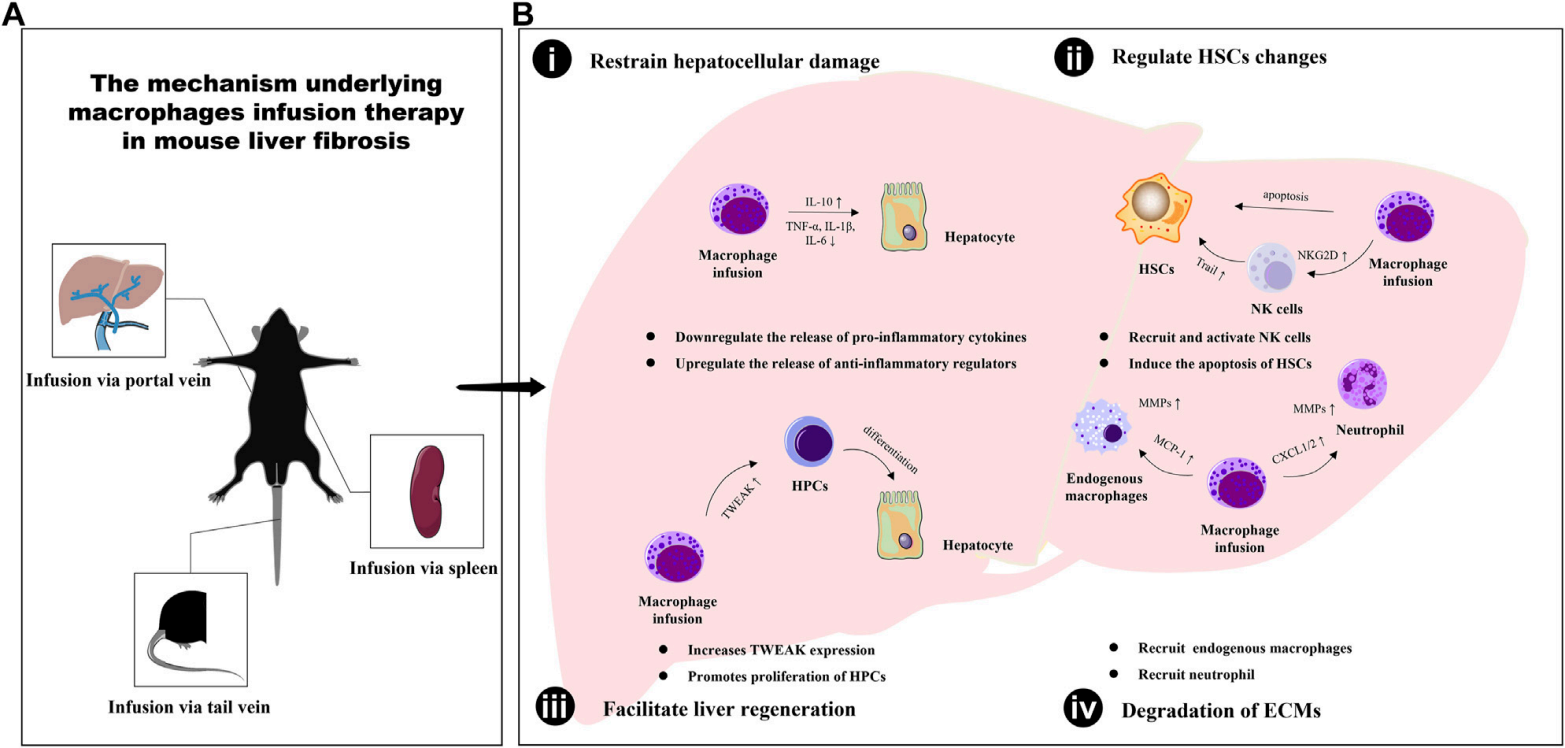
The mechanism underlying macrophages infusion therapy in mouse liver fibrosis. **(A)** The different types of macrophages are transfused into mice in three ways: tail vein injection, portal vein injection and spleen injection. **(B)** The infusion of macrophages has been shown to play a critical role in ameliorating liver fibrosis through enabling inter-population communication serves to restrain hepatocellular damage **(i)**, regulate HSCs changes **(ii)**, facilitate liver regeneration **(iii)** and promote extracellular matrix degradation **(iv)**.

The infusion of macrophages has been shown to play a critical role in ameliorating liver fibrosis through crosstalk with various cell types in the liver, including hepatocytes, hepatic stellate cells (HSCs), natural killer (NK) cells, endogenous macrophages, neutrophils, and hepatic progenitor cells (HPCs). This inter-population communication serves to facilitate liver inflammation mediation, promote collagen degradation, and enhance liver regeneration, ultimately contributing to the regression of hepatic fibrosis (as illustrated in Figure 1).

### 4.1 Inhibition of hepatocellular injury

The liver is mainly comprised of hepatocytes, which make up around 80% of its volume and perform crucial functions such as protein synthesis and energy metabolism regulation (Blouin et al., 1977). Liver fibrosis is a consequence of liver cell damage and repair. To effectively treat liver fibrosis, it is imperative to prevent hepatocyte death by addressing the root cause of injury. Various studies have suggested that there is communication between hepatic macrophages and hepatocytes. Macrophages can help alleviate liver fibrosis by eliminating dead hepatocytes and reducing inflammation in the liver (Triantafyllou et al., 2018; Shi et al., 2022). In a liver fibrosis mouse model, the transfer of differentiated macrophages from bone marrow via tail vein infusion promoted increased levels of anti-inflammatory factors such as IL-10, which protected against liver damage caused by inflammation (Thomas et al., 2011). The infused KCs also contributed to a reduction in inflammatory cells in the portal areas of the liver, while levels of pro-inflammatory factors IL-1β, IL-6, and TNF-α were decreased. An increase in serum albumin levels indicated an improvement in liver function (Li and He, 2021). Notch signaling has been found to regulate inflammatory responses. Furthermore, genetic modification of BMDMs via tail vein infusion protected hepatocytes from damage in a hepatic ischemia-reperfusion injury model, with an upregulation of hepatic expression of Notch1 and a downregulation of IL-1β and TNF-α (Ke et al., 2010; Huang et al., 2014). Additionally, macrophage infusion was found to protect mice from acetaminophen-induced acute liver injury (Merlin et al., 2016; Starkey Lewis et al., 2020). These findings highlight the potential of macrophage therapy in protecting hepatocytes from damage and reducing inflammation in the liver, which could ultimately lead to the prevention and treatment of liver fibrosis.

### 4.2 Regulate HSCs changes

Liver fibrosis is underpinned by the myofibroblastic trans-differentiation of hepatic stellate cells (HSCs) in response to liver injury, with α-SMA being a marker for activated HSCs. To prevent the progression of hepatic fibrosis, it is crucial to reduce HSC activation and transition or promote their senescence and apoptosis (Kong et al., 2012; Wang et al., 2020), which can occur following macrophage infusion. Remarkably, a significant decrease in the number of α-SMA-positive myofibroblast cells was observed in fibrotic mice treated with ESDMs, particularly those treated with the higher dose of 20 × 10^6^ cells (Haideri et al., 2017). Thomas et al. (Thomas et al., 2011) previously demonstrated that transplantation of 1 × 10^6^ BMDMs had a therapeutic effect, with α-SMA staining falling to 40% of control after 7 days. However, the decrease in myofibroblasts was no longer statistically significant after 1 month, indicating that the reduction in the myofibroblast population occurs soon after BMDMs delivery. Similar findings were reported in other studies, where BMDMs (LPS/IFN-γ) significantly reduced α-SMA and Desmin positive signals. α-SMA and TUNEL double staining further demonstrated that HSC apoptosis was an early event after macrophage infusion (Ma et al., 2017). Additionally, Li et al. (Li and He, 2021) found that KCs infusion similarly reduced the expression of α-SMA and decreased the expression of TGF-β, a master pro-fibrogenic cytokine associated with HSCs activation and liver fibrosis. It is worth noting that the changes in HSCs are influenced by other liver cells, including NK cells (Ping et al., 2023). It was observed that the infusion of BMDMs (LPS/IFN-γ) stimulated a significant increase in the number of NK cells, suggesting their recruitment. Moreover, NKG2D expression on NK cells in the liver was upregulated, along with high levels of TNF-related apoptosis inducing ligand (TRAIL)which mediated the induction of HSC apoptosis (Ma et al., 2017; Roehlen et al., 2020). This result indicated that the infused BMDMs (LPS/IFN-γ) increased the recruitment and activation of NK cells, ultimately contributing to the amelioration of fibrogenesis through TRAIL-mediated HSC apoptosis. These findings suggest that macrophage therapy may be a promising approach in reducing HSC activation and promoting their senescence and apoptosis, ultimately leading to the prevention and treatment of liver fibrosis.

### 4.3 Promote degradation of ECMs

The degradation of ECM components is controlled by the equilibrium between matrix metalloproteinases (MMPs) and their endogenous inhibitors, tissue inhibitors of MMPs (TIMPs). This balance is a predominant factor in the regression of liver fibrosis (Xu et al., 2004). It is widely acknowledged that MMPs are stored in macrophage and neutrophil granules. BMDMs therapy could stimulate the upregulation of monocyte chemotactic protein-1 (MCP-1), a macrophage chemokine, and CXCL1 and CXCL2, neutrophil chemokines, respectively (Thomas et al., 2011). MCP-1 belongs to the CC chemokine subfamily and binds to the CCR2 receptors of monocytes, while CXCL1 and CXCL2 may bind to the CXCR2 neutrophil surface receptor (Zlotnik and Yoshie, 2012). The interplay between these cytokines may play an instrumental role in the recruitment of endogenous macrophages and neutrophils. In this study, both cells contributed to the expression of MMP-9 and MMP-13 in the liver, leading to improved liver fibrosis, respectively. BMDMs (LPS/IFN-γ) infusion led to higher levels of endogenous macrophage infiltration than naïve BMDMs. This may be attributed to the elevated macrophage chemokines CCL2 and CCL3. In BMDMs (LPS/IFN-γ) treated fibrotic mouse livers, the expression of MMP-2, MMP-9, and MMP-13 was significantly increased, while the expression of TIMP-1 and TIMP-2 was decreased. Interestingly, BMDMs (LPS/IFN-γ)-recruited macrophages had a Ly6C^low^ restorative phenotype characterized by the authors as possessing high levels of MMPs and more phagocytic characteristics (Ma et al., 2017). Similar results were obtained through KCs infusion therapy, where the mRNA levels of MMP-2, MMP-9, and MMP-13 increased in endogenous F4/80^+^ cells from the liver after the infusion of KCs (Li and He, 2021). The above results approve that macrophage therapy may promote the expression of MMPs and contribute to the degradation of ECM components, ultimately leading to the regression of liver fibrosis.

### 4.4 Facilitate liver regeneration

In addition to relying on fiber-degrading, the reversal of hepatic fibrosis involves the restoration of hepatocyte numbers and function. Hepatic progenitor cells (HPCs) are stem cells that possess bipotentiality and can express markers of both hepatocytes and cholangiocytes. It was previously believed that HPCs were located at the level of Canals of Hering and differentiated into hepatocyte-like cells to aid in liver regeneration (Pu et al., 2023). Thomas et al. (Thomas et al., 2011) found that the mRNA levels of the HPCs marker cytokeratin-19 significantly increased in BMDMs recipients 3 days after delivery, with a 55% increase observed compared to control recipients. This suggests that BMM delivery may positively impact HPCs proliferation and differentiation. By day 7, there was a periportal expansion of pancytokeratin (PanCK)-positive HPCs in BMDMs recipients, indicating that BMDMs delivery may promote the differentiation of HPCs into mature hepatocytes (Thomas et al., 2011). Interestingly, a study reported an increase in PanCK^+^ cells after the infusion of BMDMs into normal mice via tail vein (Bird et al., 2013). In CCl_4_-injured animals who received both doses of ESDMs, there was a significantly higher number of PanCK-positive cells compared to control injured livers (Haideri et al., 2017). Moreover, the infusion of BMDM (LPS/IFN-γ) has been shown to improve hepatocyte proliferation and liver function in fibrotic liver disease (Ma et al., 2017). Taken together, these findings suggest that BMDM-based therapies may hold promise as a potential treatment for liver diseases by enhancing the proliferation and differentiation of HPCs and other hepatic progenitor cells.

Tumor necrosis factor-like weak inducer of apoptosis (TWEAK) is a member of the TNF superfamily that regulates mesenchymal progenitor cells through its receptor, Fibroblast growth factor-inducible 14 (Fn14). TWEAK usually stimulates the proliferation of hepatic progenitor cells (HPCs), which are stem cells that can differentiate into hepatocytes and aid in liver regeneration (Ko et al., 2019). Interestingly, endogenous macrophages have been identified as a major cell source of TWEAK during chronic liver injury (Bird et al., 2013). In studies involving BMDMs inoculated animals, the expression of TWEAK indicated significant proliferation of macrophage-mediated HPCs (Thomas et al., 2011). Additionally, Bird et al. (Bird et al., 2013) confirmed that the activation of ductular reaction following macrophage infusion therapy is dependent on TWEAK signaling. Furthermore, the infusion of HMDMs into a mouse hepatic fibrosis model also causes significant changes in TWEAK expression in the liver (Moore et al., 2015).

Other research has also confirmed the significance of macrophages in facilitating liver regeneration. For instance, in rats subjected to portal vein ligation, infusion of CD86^+^ macrophages of a specific type has been demonstrated to accelerate the liver regeneration response (Zhao et al., 2021). This discovery highlights the crucial role played by macrophages in promoting liver regeneration following injury. In a separate investigation, a mouse model was established to study liver injury and regeneration via partial hepatectomy, whereby BMDM (IL-4) were injected to aid in hepatocyte proliferation. The key lipid S1P was discovered to facilitate the infusion of BMDMs (IL-4), thereby promoting hepatocyte proliferation after hepatectomy. Notably, this study also revealed that infusion of BMDMs (LPS) led to hepatocyte apoptosis after hepatectomy (Sun et al., 2021). These results indicate that the functional phenotype of macrophages is crucial in regulating liver regeneration. To completely comprehend the mechanisms behind macrophage-mediated liver regeneration and to create novel therapies for liver injury and disease, more extensive research in this field is necessary.

## 5 Clinical trial of autologous macrophage therapy for liver cirrhosis

A common feature in patients with liver cirrhosis is a decrease or loss of liver macrophages (Ramachandran et al., 2019). However, the exact reasons for this are still unclear, and some hypotheses suggest that it may be due to the loss of essential cell-cell circuits, such as a loss of stellate cell genes leading to loss and inhibition of macrophages (Zhao et al., 2022). Despite the fact that there have been several large and well-conducted clinical trials exploring autologous cells therapy for liver cirrhosis, research on macrophages has been minimal. However, macrophages have been proven to be effective in treating liver fibrosis under Good Manufacturing Practice (GMP) compliant conditions, and this provides a new direction for the treatment of liver fibrosis in the future (Fraser et al., 2017). In 2019, a first-in-human, phase 1 dose-escalation trial of autologous macrophage therapy in nine adults with cirrhosis was conducted (Moroni et al., 2019). The results of this study showed that in 6 out of 9 patients, reductions in model for end-stage liver disease (MELD) score were observed. Moreover, several non-invasive measures of liver fibrosis improved following macrophage infusion, including transient elastography, serum ELF score (Friedrich-Rust et al., 2010) and the collagen turnover markers the N-terminal propeptide of type III collagen and type III collagen (Moroni et al., 2019). These findings highlight the potential antifibrotic effect of autologous monocyte-derived macrophage infusion in cirrhosis, confirming the safety, feasibility and maximum achievable dose of autologous macrophages. Currently, a randomized controlled phase 2 study is gradually being carried out to examine the efficacy of autologous macrophage therapy in improving liver function, noninvasive fibrosis markers and other clinical outcomes in patients with compensated cirrhosis (Brennan et al., 2021). This trial will provide the first high-quality examination of the efficacy of autologous macrophage therapy in treating liver cirrhosis. If successful, this therapy could offer a promising treatment option for patients suffering from liver cirrhosis.

## 6 Conclusion

In summary, recent studies have shown the potential of using exogenous macrophages to treat liver fibrosis, but there are still several challenges, which including identifying the most suitable source of macrophages, optimizing the dosage and frequency of macrophage transplantation, and improving the survival rate and function of macrophages, need to be addressed. To overcome these challenges, further research is needed to investigate the underlying mechanisms of exogenous macrophages in treating liver fibrosis and to optimize their use in clinical practice. By doing so, we can better understand how to harness the potential of exogenous macrophages as a promising treatment option for patients suffering from liver fibrosis.
